# A non-parametric effect-size measure capturing changes in central tendency and data distribution shape

**DOI:** 10.1371/journal.pone.0239623

**Published:** 2020-09-24

**Authors:** Jörn Lötsch, Alfred Ultsch

**Affiliations:** 1 Institute of Clinical Pharmacology, Goethe—University, Frankfurt am Main, Germany; 2 Fraunhofer Institute of Molecular Biology and Applied Ecology—Project Group Translational Medicine and Pharmacology (IME-TMP), Frankfurt am Main, Germany; 3 DataBionics Research Group, University of Marburg, Marburg, Germany; Rutgers University, UNITED STATES

## Abstract

**Motivation:**

Calculating the magnitude of treatment effects or of differences between two groups is a common task in quantitative science. Standard effect size measures based on differences, such as the commonly used Cohen's, fail to capture the treatment-related effects on the data if the effects were not reflected by the central tendency. The present work aims at (i) developing a non-parametric alternative to Cohen’s d, which (ii) circumvents some of its numerical limitations and (iii) involves obvious changes in the data that do not affect the group means and are therefore not captured by Cohen’s d.

**Results:**

We propose "Impact” as a novel non-parametric measure of effect size obtained as the sum of two separate components and includes (i) a difference-based effect size measure implemented as the change in the central tendency of the group-specific data normalized to pooled variability and (ii) a data distribution shape-based effect size measure implemented as the difference in probability density of the group-specific data. Results obtained on artificial and empirical data showed that “Impact”is superior to Cohen's d by its additional second component in detecting clearly visible effects not reflected in central tendencies. The proposed effect size measure is invariant to the scaling of the data, reflects changes in the central tendency in cases where differences in the shape of probability distributions between subgroups are negligible, but captures changes in probability distributions as effects and is numerically stable even if the variances of the data set or its subgroups disappear.

**Conclusions:**

The proposed effect size measure shares the ability to observe such an effect with machine learning algorithms. Therefore, the proposed effect size measure is particularly well suited for data science and artificial intelligence-based knowledge discovery from big and heterogeneous data.

## Introduction

Calculating the extent of treatment effects or group differences is a common task in quantitative biomedical science [1]. Effect sizes allow quantification of the influence of independent variables (features, e.g. pathophysiological processes, risk factors, interventions, treatments) on dependent variables (e.g. subgroups, disease diagnoses) [2] inclusively a ranking among those features [3]. Although several different measures have been proposed, their use in biomedical research remains an active research topic [3, 4].

Most commonly used effect measures are difference-based, i.e. the effect is proportional to a standardized difference between groups in the central tendency of a feature, such as in Cohen's d [5], which is a widely used effect size measure. In fact, PubMed search for “Cohen's d” on June 26, 2020, resulted in 4,199 hits. Cohen's d was used, for example, to compare the effects of various factors on pain [6, 7], or to compare job search methods used by people at risk of poverty and social exclusion [8]. Since the Cohen's d is also one of the most widely used measures in meta-analyses (e.g. [9, 10]), it was used as the basis for the present proposal of a novel effect size measure. The motivation behind this proposal was (i) to develop a non-parametric alternative to Cohen’s d, which (ii) circumvents some of its numerical limitations and (iii) involves obvious changes in the data that do not affect the group means and are therefore not captured by Cohen’s d.

**Fig 1:**
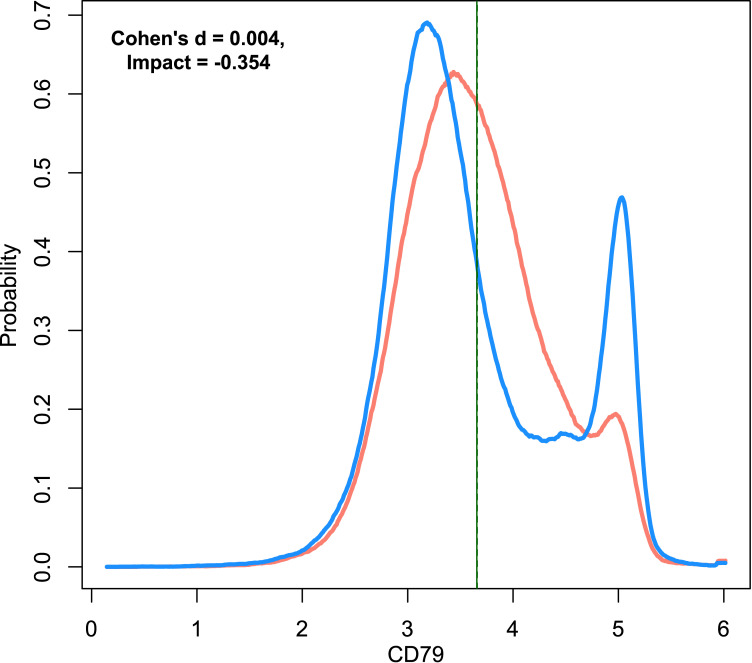
Example data set 5 showing the expression of the B-cell antigen receptor complex-associated protein CD79 in patients with B-cell lymphoma (n = 127397, blue line) and healthy controls (n = 131032, red line). A bimodal distribution of CD79 in flowcytometry-derived data has been reported as a typical observation in samples from patients with hematological malignances [11]. The density distribution is presented as probability density function (PDF), estimated by means of the Pareto Density Estimation (PDE. The perpendicular lines indicate the means for both data subsets (solid and dashed green lines). The means are numerically identical but have been optically separated by one pixel. The figure has been created using the R software package (version 3.6.3 for Linux; http://CRAN.R-project.org/ [25]) using our R package “ImpactEffectsize” (https://cran.r-project.org/package=ImpactEffectsize).

One of the above-mentioned limitations of purely difference-based effect measures such as Cohen’s d remains that the estimated effect must be zero with unchanged central tendency. This sometimes stands in sharp contrast to the visualization of biomedical data (Fig 1). In this example, a bimodal expression of the B-cell antigen receptor complex-associated protein CD79 in patients with lymphoma changes visibly the distribution of the maker in a flowcytometric data set, but the means are almost the same in patients and controls. Changes in the data distribution seems a typical finding for CD79 also in other hematological malignancies such as B-cell chronic lymphocytic leukemia [11]. Similar observations apply to further markers such as in adult common acute lymphoblastic leukemia where immunophenotypically distinct subpopulations are found with bimodal marker expression or populations with broad marker expression [12]. In such data, for the latter variables Cohen’s d might indicate disease effects while the first type of markers might escape inclusion in positive feature sets.

We propose a novel effect size measure, called "Impact”, which captures effects that change the central tendency of the data as well as effects that change the shape of the data distribution. This may increase its usefulness as a generic effect size measure for the initial exploration of large and extensive data sets and provide a unifying description of effects on many different and heterogeneously distributed variables.

## Methods

### “Impact”effect size measure

#### Design criteria

The design criteria for the proposed measure of effect size were, first, that the measure should not be parametric. Second, the measure should be invariant to the scaling and transformation of the data. Third, if the changes in the shape of the probability distributions between subgroups are negligible, it should reflect only the change in the central tendency and vice versa. Fourth, changes in the probability distributions should be recorded. Fifthly, the measure should be numerically stable, especially when the variances of the data set or its subgroups disappear. Sixthly, the measurement of effect size should generally be consistent with the estimation using Cohen's d.

#### Definitions

*Impact(X1*, *X2)* defines an effect size based on the difference in central tendency between two groups or experimental conditions where *X1* and *X2* are subgroups of a data set *X* = {*X*1 ⋃ *X*2}. *Impact(X1*, *X2)* is defined as zero, if the hypothesis that *X1* and *X2* are identical cannot be rejected by a Kolmogorov Simonov test [13]. Otherwise, *Impact(X1*, *X2)* is the sum of two separate measures of effects sizes comprising (i) a difference-based effect size measure implemented as the change in the central tendency of the group-specific data, *CTdiff(X1*,*X2)*, normalized to pooled variability and (ii) a data distribution shape based effect size measure implemented as the difference in probability density of the group-specific data, called morphic difference *MorphDiff(X1*,*X2)*. That is,

Impact(X1,X2)=CTDiffWeight·DirCT·CTdiff(X1,X2)+(1−CTDiffWeight)∙DirMorph∙MorphDiff(X1,X2) Eq 1

*Difference-based effect size component*. The central tendency difference *CTdiff(X1*, *X2)* is calculated as

$$CTdiff=\frac{\left|median(X2)-median(X1)\right|}{GMD(X1,X2)}$$ Eq 2

with *GMD(X1*, *X2)* denoting the expected value of absolute inner differences in *X*. *GMD* is the Gini’s mean difference (GMD [14]) providing an appropriate measure of the variability of non-normal distributions [10], which is defined as

$$GMD(\xi )=\frac{1}{n^{2}}\sum_{i=1}^{n}\sum_{j=1}^{n}\left|\xi_{i}-\xi_{j}\right|,n=|\xi |,\xi =\{\xi i\}$$ Eq 3

The pooled variability, *GMD(X1*, *X2)*, is defined using GMD as

$$GMD(X1,X2)=\left\{\begin{array}{c} \sqrt{\left(\frac{{GMD(X1)}^{2}+{GMD(X2)}^{2}}{2}\right)}\;if\;Var(X1)>0\;and\;Var(X2)>0\\ GMD(X)\;if\;(Var(X1)=0\;or\;Var(X2)=0)\;and\;Var(X)>0\\ \varepsilon \;if\;Var(X)=0\;with\;0<\varepsilon \ll 1 \end{array}\right\}$$ Eq 4

The direction of *CTdiff* is defined as

$$DirCT=\left[\begin{array}{cc} -1 & median(X2)<median(X1)\\ 1 & otherwise \end{array}\right]$$ Eq 5

Finally, a weighting factor is assigned to the difference component, defined as

CTDiffWeight=min(CTdiff,2)/2 Eq 6

accommodating that the shape-based component of the effect size has a maximum value of 2 as specified below.

*Data distribution shape-based effect size component*. The second summand of *Impact(X1*,*X2)* (see Eq 1) is the so-called morphic difference *MorphDiff(X1*,*X2)*. It describes the differences in the probability distributions of *X1* and *X2*:

MorphDiff(X1,X2)=∙∫(|pdf(X2)−pdf(X1)|) Eq 7

where *pdf(ξ)* denotes the probability distribution of data. Empirically, *pdf(ξ)* can be calculated by a suitable estimation such as the Pareto density estimation (PDE) [15]. The direction of *MorphDiff* is defined as

$$DirMorph=\left[\begin{array}{cc} -1 & momentum(X2)<momentum(X1)\\ 1 & otherwise \end{array}\right]$$ Eq 8

$$with\;momentum(\xi )=\frac{1}{L(|\xi |)}\int L(\xi )$$ Eq 9

where L*(ξ)* denotes the log-modulus transformation of the data that provides a zero-invariant log transformation preserving the sign of data [16], which is defined as L(ξ) = sign(ξ) * log(|ξ| + 1).

### Data sets

To evaluate the properties of the proposed effect size measure, to compare its results with those of Cohen's d and to assess its usefulness for two-class comparison problems, artificially generated and empirically collected biomedical data sets were used. Basic descriptive statistics of the data sets are shown in Table 1.

**Table 1:** Descriptive statistics of the used data sets.

Data set	Subset	N		Mean		SD		Median	
		Group 1	Group 2	Group 1	Group 2	Group 1	Group 2	Group 1	Group 2
**1**	1	1000	1000	0.036	-0.026	4.156	4.13	0.137	-0.073
	2	1000	1000	-2.327	-2.335	3.326	3.41	-2.258	-3.645
	3	1000	1000	2.327	2.335	3.326	3.41	2.258	3.645
	4	1000	1000	-0.012	0.02	0.974	4.033	-0.006	-0.113
	5	1000	1000	1.896	1.876	1.861	1.904	1.351	1.864
	6	1000	1000	-1.896	-1.932	1.861	0.925	-1.351	-1.936
**2**	1	100	100	1	2	1	2	1	2
	2	100	100	1	1	0	0	1	1
	3	100	100	1	2	0	0	1	2
	4	100	100	3	3	1.4	1.4	3	3
	5	100	100	49.7	47.7	30.3	28.4	46.5	51.5
	6	100	100	497.1	477.3	302.9	284.1	465	515
**3**	Dynamically created as described in the data sets section								
**4**	1	1000	1000	0.008	-0.069	1.007	1.001	0.007	-0.077
	2	1000	1000	-0.015	-0.002	0.992	0.984	-0.049	-0.005
	3	1000	1000	0.015	-0.012	0.983	1.008	0.033	-0.027
	4	1000	1000	-0.015	0.027	1.027	1.059	-0.052	0.029
	5	1000	1000	0.011	-0.025	0.96	0.995	0.015	-0.023
	6	1000	1000	0.025	-0.012	1	0.979	0.032	0.027
	7	1000	1000	0.036	0.031	0.98	0.989	0.044	0.009
	8	1000	1000	-0.025	0.052	1.011	0.998	-0.032	0.049
	9	1000	1000	0.04	-0.05	1.006	1.06	0.006	-0.09
	10	1000	1000	-0.014	-0.031	1.008	0.974	0.026	-0.036
	11	1000	1000	-0.006	3.014	1.011	0.989	0.035	2.987
	12	1000	1000	-0.014	4.011	0.964	1.006	-0.036	3.961
	13	1000	1000	0.055	5.061	1.021	1.026	0.038	5.049
	14	1000	1000	-0.005	6.009	1.012	0.973	0	6.023
	15	1000	1000	-0.003	7.01	0.995	0.954	0.011	7.013
	16	1000	1000	0.032	0	1.04	3.263	0.072	0
	17	1000	1000	-0.004	0	0.993	4.127	-0.006	0
	18	1000	1000	-0.003	0	0.972	5.175	-0.032	0
	19	1000	1000	-0.03	0	0.984	6.059	-0.022	0
	20	1000	1000	0.055	0	1.049	7.058	0.028	0
**5**	1	131032	127397	3.66	3.66	0.684	0.822	3.59	3.44
**6**	1	1494	1302	4.79	4.8	0.28	0.29	4.84	4.82
	2	1494	1302	5	4.97	0.23	0.4	5.04	5.04
	3	1494	1302	3.96	3.84	0.42	0.47	3.88	3.74
	4	1494	1302	3.32	3.3	1.01	0.83	3.56	3.51
	5	1494	1302	3.74	3.68	0.31	0.4	3.78	3.75
	6	1494	1302	3.43	3.15	0.37	0.52	3.5	3.14
	7	1494	1302	3.66	3.6	0.44	0.51	3.66	3.64
	8	1494	1302	2.89	2.9	0.53	0.69	2.81	2.67
**7**	1	4687	835	-0.000776	0.0088	0.01623	0.0603	0	0.037

#### Artificially generated data sets

**Fig 2:**
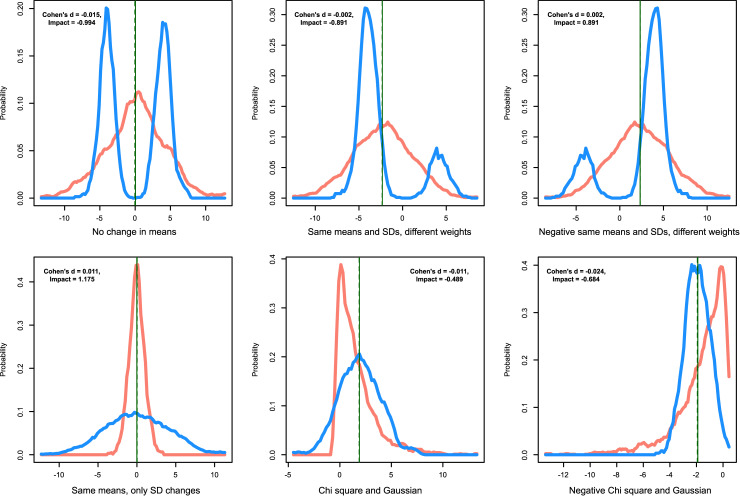
Artificial data where the two groups (red and blue) display no differences in mean but possess clearly different data distributions (data set 1). The axis legend of the abscissa indicates the kind of data distributions underlying the groups. The density distribution is presented as probability density function (PDF), estimated by means of the Pareto Density Estimation (PDE [15]). The perpendicular lines indicate the means for both data subsets (solid and dashed green lines). The means are numerically identical but have been optically separated by one pixel. The figure has been created using the R software package (version 3.6.3 for Linux; http://CRAN.R-project.org/ [25]) using our R package “ImpactEffectsize” (https://cran.r-project.org/package=ImpactEffectsize).

**Data set 1** (Fig 2) was used to show the differences between Cohen's d and “Impact”in brief. This data set consists mainly of subsets, with no differences in the central tendency, but a considerable change in the shape of the distributions of the subsets. The data set was created with the property that both groups have the same means. Six subsets were created with n = 2000 points, unless otherwise specified n_1_ = 1000 and n_2_ = 1000. The first subset X_1_ had the property that the means and variances were the same in both groups. The effect of an assumed treatment is that a standard unimodal normal distribution (N(0,1)) is changed to a bimodal distribution (N(-5,1), N(5,1)) containing 50% of the data in each mode. The second and third subsets were essentially the same, but contained 80% of the data in one mode and 20% in the other mode and *vice versa*. The data subset three X_3_ was the data set two X_2_, mirrored on the y axis: X_3_ = -X_2_. The fourth subset of data consisted of a standard normal distribution for X_1_ and a Gaussian distribution with the same mean but with a standard deviation of four (N(0,4)). The fifth (X_5_) and sixth (X_6_) data subset consisted of a normal distribution in one group and a chi-square distribution in the other group, with the same mean as the Gaussian distribution, and with X_6_ = —X_5_.

**Fig 3:**
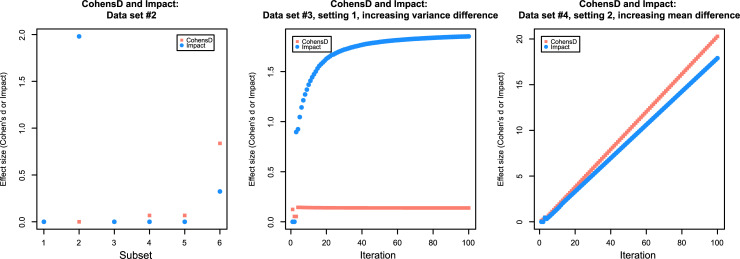
Dot plots of the effect sizes expressed as Cohen’s d and “Impact”calculated for artificially created data sets (data sets 2 and 3) comprising subsets of each of two groups (red and blue) with identical sizes of n = 100. **Left panel:** Data set 2 in which subsets X_1_ had only one value (x_i_ = 1) in all cases and groups. In the second subset X_2_, one group contained always the value of x = 1 and the other group always the value of x = 2. In the third subset X_3_, the sequence [1,…,5] was repeated 20 times, so that both groups were identical and contained the even numbers from 1 to 5 at equal frequencies. In the fourth subset X_4_, the groups were created by random sampling with replacement from sequences [1,…,100], independently for each group. The fifth subset was X_5_ = 10 · X_4_ to study the scale-independency of “Impact”. In the sixth subset X_2_ group 1 contained always a value of 1 while group 2 was created as 100 values from a normal distribution with mean = 2 and standard deviation = 2. **Middle panel:** Data set 3 with several iteratively created data subsets with groups with the same mean but increasing variance in one but not the other group. **Right panel:** Data set 3 with several iteratively created data subsets with groups with the same variance but increasing mean in one but not the other group. The figure has been created using the R software package (version 3.6.3 for Linux; http://CRAN.R-project.org/ [25]) using our R package “ImpactEffectsize” (https://cran.r-project.org/package=ImpactEffectsize).

**Data set 2** (Fig 3, left panel) was created to test some extreme scenarios. For some of these scenarios it is known that effect size measures based upon Cohen's d would fail. It comprised six subsets with n = 200 points, unless otherwise specified n_1_ = 100 and n_2_ = 100. The first subset X_1_ had only one value (x_i_ = 1) in all cases and groups. In the second subset X_2_, one group contained always the value of x = 1 and the other group always the value of x = 2. In the third subset X_3_, the sequence [1,…,5] was repeated 20 times, so that both groups were identical and contained the even numbers from 1 to 5 at equal frequencies. In the fourth subset X_4_, the groups were created by random sampling with replacement from sequences [1,…,100], independently for each group. The fifth subset was X_5_ = 10 · X_4_ to study the scale-independency of “Impact”. In the sixth subset X_2_ group 1 contained always a value of 1 while group 2 was created as 100 values from a normal distribution with mean = 2 and standard deviation = 2.

**Data set 3** (Fig 3, middle and right panel) was created to separately and systematically test the two components of “Impact”, i.e. the difference in the central tendency or the shape of the data distribution. It comprised several dynamically created data subsets with n = 200 points and n_1_ = 100 and n_2_ = 100. In a first setting, 100 normally distributed data sets were created in which the two groups had the same mean (m = 1) but the standard deviation was constant at σ = 1 in one group but increased as σ = 1,…,100 in the other group. The second setting was the opposite, i.e., here the standard deviations were kept constant at σ = 5 but in one group the mean was constant at m = 5 while in the other group the mean increased as m = 1,…,100.

**Fig 4:**
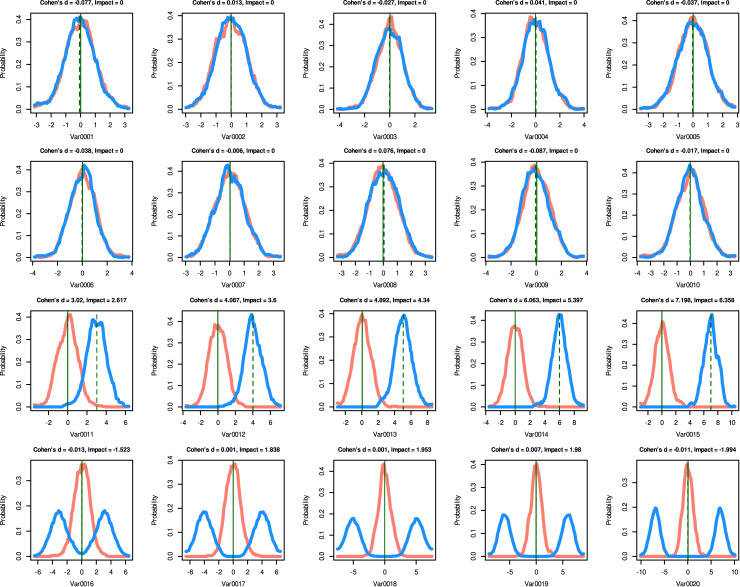
Artificially created data comprising two groups (red and blue) with sizes of n = 1000 and d = 20 variables (data set 4). Of these variables, in var0001 –var0010 the means and variances were randomly jittered between the two groups (upper two lines of panels), in variables va0011 –var0015 the means differed substantially between groups (third line of panels), and in var0016 –var0020 the groups had the same mean but one group the data was spilt into two distinct modes whereas in the other group the data varied around the mean (bottom line of panels). The density distribution is presented as probability density function (PDF), estimated by means of the Pareto Density Estimation (PDE [15]). The perpendicular lines indicate the means for both data subsets (solid and dashed green lines). The figure has been created using the R software package (version 3.6.3 for Linux; http://CRAN.R-project.org/ [25]) using our R package “ImpactEffectsize” (https://cran.r-project.org/package=ImpactEffectsize).

**Data set 4** (Fig 4) was created to examine the properties of Cohen's d compared to “Impact” including correlation analyses between the two (Pearson correlation [17]). In addition, the data set was used to comparatively assess the effect sizes measures in a machine learning context (see chapter experiment). It contained d = 20 variables (characteristics) with group sizes of n_1_, n_2_ = 1000. Ten variables were created as standard normal distributions (N(0,1)) using the same random number generator for all data subsets. The differences in these subsets should give values around zero in all effect measures. Five variables consist of a subset drawn from a standard normal distribution, the other subsets were drawn from a Gaussian distribution with mean = 3,…,7 and unit variance. For these variables, the effect size measures should be considerable and proportional to the difference in mean values. The last five characteristics consist of a subset drawn from a standard normal distribution, the other subsets were drawn from a bimodal distribution, so that the mean value of these subsets is zero, i.e. no change in the mean values between the two subsets, but with considerable and increasing changes in their probability distribution. An appropriate sorting of the characteristics in this data set in descending order of absolute effect size should be 15,…,11 (i.e. differences in the central tendency), then the variables numbered 20,…,16 (differences in pdf) and then any order of variables 1 to 10 (the subsets differ only by minor noise in the data).

#### Biomedical data sets

**Data set 5** served as the introductory example (Fig 1). It has been taken from a real-live data set created by flowcytometric analyses of blood samples from patients with B-cell lymphoma (n = 127397) and from healthy controls (n = 131032). It contains expression values of the cluster of differentiation marker 79 (CD79) that in association with surface immunoglobulin constitutes the B-cell antigen receptor complex and plays a critical role in B-cell maturation and activation. A bimodal distribution of CD79 in flowcytometry-derived data has been reported as a typical observation in samples from patients with hematological malignances [11].

**Fig 5:**
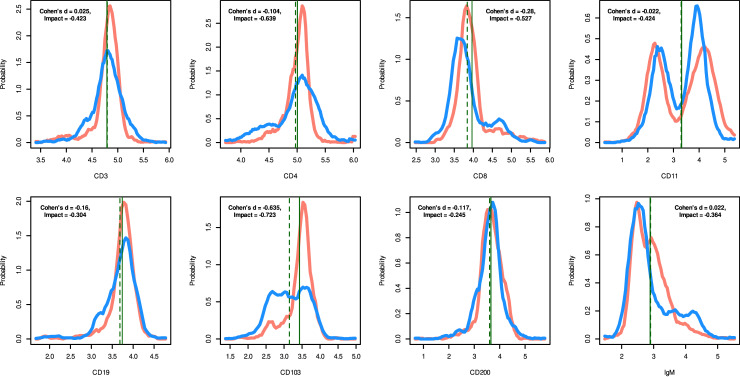
Biomedical data of a hematological context comprising a flow cytometry-based lymphoma makers CD3, CD4, CD8, CD11, CD19, CD103, CD200 and IgM comprising of one subset of n = 1,494 cells from healthy subjects (red) and a second set of n = 1,302 cells from lymphoma patients (blue) (data set 6). The density distribution is presented as probability density function (PDF), estimated by means of the Pareto Density Estimation (PDE [15]). The perpendicular lines indicate the means for both data subsets (solid and dashed green lines). The figure has been created using the R software package (version 3.6.3 for Linux; http://CRAN.R-project.org/ [25]) using our R package “ImpactEffectsize” (https://cran.r-project.org/package=ImpactEffectsize).

**Data set 6** (Fig 5) was used to include a broader selection of markers in a hematological context. It comprises eight different immunological markers associated with the diagnosis of lymphoma from a flow cytometric panel-based blood analysis. The measurements consist of a subset of n = 1,494 cells from healthy volunteers and a second subset of n = 1,302 cells from lymphoma patients. Cell surface molecules that provide targets for the immunophenotyping of the cells, i.e. CD3, CD4, CD8, CD11, CD19, CD103, CD200 and IgM, were used as measurement parameters.

#### Non-biomedical data set

**Fig 6:**
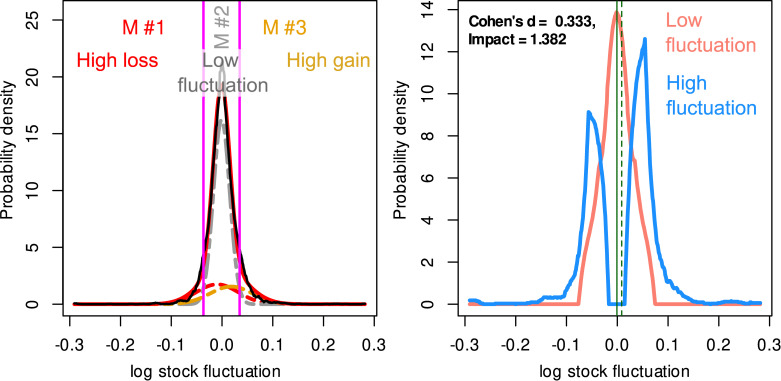
Logarithmic returns of n = 10 US stocks (data set 7). **Left panel:** Fit of a GMM with M = 3 modes. The distribution of the data is shown as probability density function (PDF) estimated by means of the Pareto density estimation (PDE; black line). The GMM fit is shown as a red line and the M = 3 single mixes are indicated as differently colored dashed lines (M#1,…,M#3). The Bayesian boundaries between the Gaussians are indicated as vertical magenta lines. **Right panel:** Effect sizes between the stocks with small returns (Gaussian mode #2 in the left panel) versus the stocks with substantial returns (Gaussian modes #1 or #3 for high loss or high gain, respectively. The figure has been created using the R software package (version 3.6.3 for Linux; http://CRAN.R-project.org/ [25]) using our R packages “ImpactEffectsize” (https://cran.r-project.org/package=ImpactEffectsize) and “AdaptGauss” (https://cran.r-project.org/package=AdaptGauss [19]).

**Data set 7** (Fig 6) originates from economics and consists of n = 5,522 values describing the daily logarithmic returns of for 10 US stocks in the period 2005/08/08 to 2007/08/06 and has been downloaded from the Yahoo finance historical data at https://finance.yahoo.com/quote/SRNE/history. It was used to examine differences between various mathematical measures to calculate investment returns. In economic research, for stock returns, a distinction between returns with low variance from another group of returns with large variance may be the desired result, i.e. the identification of two Gaussian distributions with the same mean but different standard deviations. However, as shown previously [18], a reasonable alternative is to divide the stock rates into a group with almost no change and another group with large changes in either direction, which can be captured by a Gaussian mixture of M = 3 modes, with one dominant mode around zero and two smaller modes at the extremes (Fig 6). The number of modes was based on a likelihood ratio test indicating significant improvements of the fit from 1 to 3 modes but no further improvement with a forth node (details not shown). A fit of the data set using our interactive Gaussian Mixture modeling R library “AdaptGauss (https://cran.r-project.org/package=AdaptGauss [19]) provided a mixture with three means = [-0.009466400, -0.000913938, 0.018304123], standard deviations [0.04128130, 0.01614781, 0.04183527] and weights = [0.1806000, 0.6566203, 0.1627476]. Bayesian decision limits resulted at [-0.03662995, 0.03463264] and were used to create the group of stocks with small returns as those with values between the two Bayesian boundaries and the group with large fluctuations at the extremes.

### Experiment

From comparisons with Cohen’s d, it was hypothesized that while classical effect size measures share the failure to observe an effect in changes in the shape of the data distribution, but not in the central tendency with standard statistical analyses, “Impact” shares the ability to observe such an effect with machine-learning algorithms that usually have no problem in using the shape information for successful classification or group separation tasks.

To test this hypothesis, data set 4 was used (Fig 4). The variables 1,…,10 (top two lines of panels in Fig 4) were not considered relevant for group separation, since they are only randomly generated normal distributions with the same mean and standard deviation for both groups. The variables 11,…,15 were considered to be the most relevant information for group segregation because their means differed between groups and the standard deviations were chosen to maintain a clear group difference. Their effects were captured by values that differed significantly from zero for both Cohen's d and “Impact”. In contrast, variables 16,…,20 (bottom line of panels in Fig 4) had similar mean values between groups and differed only in the form of the distribution. These group differences were not captured by Cohen's d that by definition quantified the effects as near zero, while “Impact” provided effects clearly distinct from zero. The experiment was to test whether the groups could be separated without the variables 11,…,15. The expectation was that a standard statistical approach would be successful with the complete data set, but would fail if these variables were omitted, while their omission would have little effect on group separation when using machine learning. Hence the analogy to Cohen's d versus “Impact” in terms of detecting group differences in a data set depending on the presence of variables with group differences in the central tendency.

The statistical part of the experiment consisted of an analysis of variance for repeated measures (rm-ANOVA) with "measurement", i.e. either all 20 variables or a reduced set of 15 variables, from which the variables 11,…,15 were removed, as within-subjects factor and "group" as between-subjects factor. The α level was set at 0.05. Only the main effects and interactions were analyzed, without going into further details of post-hoc comparisons of individual variables. These calculations were performed using the SPSS software package (version 26 for Linux, IBM SPSS, IBM Corp, Armonk, NY, USA; https://www.ibm.com/analytics/spss-statistics-software).

The machine-learning part of the experiment consisted of classification tasks, i.e., algorithms were trained to separate the two groups based on the information contained in feature sets of either all 20 variables or of 15 variables when the variables 11,…,15 were removed. First, classification and regression trees (CART) [20] were created with variables as vertices, conditions on these variables as edges and classes as leaves. In the present form, the Gini impurity was used to find optimal (local) dichotomous decisions. Additionally, a random forest classifier [21, 22] was trained. This generates sets of different, uncorrelated and often very simple decision trees with conditions on features as vertices and classes as leaves. The distribution of the features is random and the classifier refers to the majority vote for class membership. These calculations were performed using the R-library "caret" (https://cran.r-project.org/package=caret [23]), together with the R-library "doParallel" (https://cran.r-project.org/package=doParallel [24]). The environment consisted of a the R software package (version 3.6.3 for Linux; http://CRAN.R-project.org/ [25]) on an Intel Core i9® (Intel Corporation, Santa Clara, CA, USA) computer running Ubuntu Linux 18.04.4 LTS 64-bit (Canonical, London, UK)).

The classification tasks were performed in 100-fold cross-validation runs using Monte-Carlo [26] resampling and data splitting into non-overlapping training (2/3 of the data) and test data (1/3 of the data). Classification performance was judged mainly by group assignment accuracy as group seizes were equal. In addition, further standard measures of classification performance were calculated, such as sensitivity, specificity, positive and negative predictive values, precision, recall [27, 28] and the F1 score [29, 30].

In the present analysis 500 decision trees were created containing *sqrt(d)* features as a standard of the R-library "caret". The default settings of the machine-learned algorithms were considered sufficient for the present demonstration purpose, and since elsewhere [31] it was found that there is no penalty for "too many" trees, the risk of overfitting was considered low. To further address possible overfitting, a negative control condition was created by randomly permuting each feature in the training data subset. It was expected that a classifier trained with these data will merely provide 50% classification accuracy, equivalent to turnings a coin.

### Implementation

The “Impact”effect size measure has been implemented in the R library "ImpactEffectsize" (https://cran.r-project.org/package=ImpactEffectsize). The effect size can be calculated with the “*Impact(Data*,*Cls)”* function. The input is expected to be a data vector, *Data*, and a bivalent integer vector of class information, *Cls*. The output consists of all values calculated when the effect size was estimated. The separate components *CTDiff*, *MorphDiff* and *GMD* are also provided, allowing separate access to the two components of “Impact”. In addition, the library provides data visualization implicitly by setting the “PlotIt” parameter in the main function call to “TRUE” or explicitly by calling the function “*plot2Densities(Data*,*Cls)*“. The user can display the distributions of the data using either the PDE as default or a standard density estimation provided as an R-core function when setting the “pde” switch to “FALSE” in the function call. The library uses additional functions provided in the R packages “Rcpp” (https://cran.r-project.org/package=Rcpp [32]), “caTools” (https://cran.r-project.org/package=caTools [33]), “matrixStats” (https://cran.r-project.org/package=matrixStats [34]) and “parallelDist” ([35] https://cran.r-project.org/package=parallelDist).

## Results

### Main results

#### Comparative analyses of Cohen’s d and “Impact”

In **data set 1**, where the two groups had the same mean value but obviously different shapes of distribution, Cohen's d was always close to d = 0. In contrast, “Impact” clearly showed substantial effects that corresponded both in size and direction of the visualizations. However, as observed in the first and forth subgroups (left panels in Fig 2), the negative sign of “Impact” seemed arbitrary in settings with symmetrical shapes of the data distribution. It is probably due to a rather random difference either in the group medians or a dominance of the distributions in the left direction. This shows how important it is to visualize the data when assessing the effect size. In these situations, using the absolute value of the effect size measure may be an appropriate option.

In **data set 2**, Cohen's d was not defined for those subsets (X_1_ –X_3_) where the groups contained only one or the same value within the group, i.e. zero variance (Fig 3 left panel). This was circumvented with “Impact”, which correctly captured the effect of different means between the groups but no variance within the groups (subset X_2_) and also captured identical groups as “Impact” = 0 in subset X_3_. Furthermore, “Impact”is scale-invariant, i.e., it gives the same value when the values of a data set are multiplied only by a constant factor (subsets X_4_ and X_5_).

In **data set 3**, the effect of the same mean value consisted of a constant Cohen's d close to zero, while “Impact” increased with increasing standard deviation in one group, i.e. with increasing shape difference of the data distribution between the groups (Fig 3). By its definition, a value of “Impact” = 2 could not be exceed only with the *MorphDiff* component, which was observed as expected. The opposite scenario, i.e. similar shape of the distribution but increasing differences in the central tendency between the groups, showed that “Impact” scaled proportionally with Cohen's d (Fig 3 right panel). Therefore, it seems appropriate to use for Impact the same limits for small, medium or large effects as generally accepted for Cohen’s d [36].

**Fig 7:**
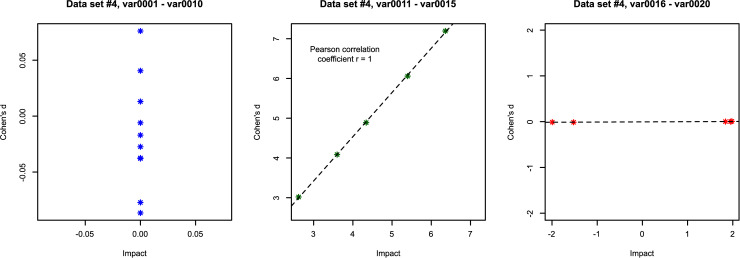
Relations between the “Impact” effect size measure and Cohen’s d in the different data scenarios of data set 4 (Fig 4). Left panel: When data of both groups are randomly generated normally distributed numbers with small between-group differences in mean and variance (var0001 to var0010 of data set 4), the effects are small, i.e., equal or close to zero. Middle panel: When differences in the group means increase (var0011 to var0015), Cohen’s d and “Impact”are correlated. Right panel: With no change in the central tendency difference but group differences only in the distribution of data. Cohen’s d takes a value of zero in these cases, “Impact”measures the effect. The dotted lines indicate linear regressions. The figure has been created using the R software package (version 3.6.3 for Linux; http://CRAN.R-project.org/ [25]) using our R package “ImpactEffectsize” (https://cran.r-project.org/package=ImpactEffectsize).

In **data set 4**, the variables var0001 to var0010 of data set 4 were obtained using a random number generator that produces standard normally distributed numbers. Therefore, the effects should be around zero which was the case for “Impact”. Cohen's d also yields absolute values less than 0.1 for these variables, which is below the proposed limit of d = 0.2 for a small effect [36], i.e., also indicates no effect at all (Fig 7 left panel). Var0011 to var0015 represent the effect on the differences in the group mean values (Fig 4 third line of panels). In this case, Cohen's d and “Impact”are perfectly correlated (r = 1; Fig 7 middle panel). The variables var0016 to var0020 of data set 4 show no change in the central tendency, while their distribution undergoes substantial changes. For all these features Cohen’s d takes a value of zero while “Impact” indicates substantial effects, which means that in contrast to Cohen’s d, “Impact” captured this kind of effect (Fig 3 right panel).

In **data set 5**, the situation in the clinical data was shown as an introductory example, where a typical effect consisting in the occurrence of a bimodal distribution of a marker was missed by Cohen's d, but recorded with “Impact” (Fig 5). **Data set 6** emphasized the benefit of “Impact” in similar biomedical data for a broader range of hemato-oncological markers (Fig 1).

In **data set 7** (Fig 6), “Impact” estimated the differences between stocks with large fluctuations and stocks in the middle of the distribution with zero or small fluctuations to be more pronounced (Impact = 1.38) than Cohen's d (d = 0.333), which in turn shows the additional emphasis of the Impact effect size measure on differences in the shape of the distribution between two data groups.

### Other results

#### Recognition of group differences in statistical and machine-learning settings

The statistical part of the experiment where an analysis of variance for repeated measures (rm-ANOVA) was used, a group difference in **data set 4** was only detected when the full set of variables was available (Table 2). This agreed with the behavior of Cohen’s d that also took values differing from d ≈ 0 only in the full feature set. The analysis of variance could not detect the group difference if only variables with the same central tendency were available, i.e. those with a group shift in the mean were omitted (Table 2) and in which Cohen’s d took values of d ≈ 0, whereas “Impact” found effects among variables of the reduced data set.

**Table 2:** Results of an analysis of variance for repeated measures (rm-ANOVA) applied onto the artificially created data set 4, comprising two groups with sizes of n = 1000 and d = 20 variables (var0001—var0020, Fig 4) representing some measurements captured in the variables of this artificial data set. The analysis of variance was designed with "measurement", i.e. either all 20 variables or a reduced set of 15 variables, from which the variables 11,…,15 were removed, as within-subjects factor and "group" as between-subjects factor.

rm-ANOVA effects	All parameters [1,…,20]			Reduced set of parameters [1,…,10,16,…,20]		
	df	F	p	df	F	p
**Measurement**	19,3796	735.878	<6.65 ∙ 10^-244^	14,2792	0.139	1
Measurement * group	19,3796	736.578	<6.65 ∙ 10^-244^	14,2792	0.228	0.999
**Group**	1,1998	917.764	3.23 ∙ 10^-166^	1,1998	0.001	0.978

The machine learning part of the experiment consisted of training two classification algorithms with the complete or reduced feature sets as used above. The training of both CART and random forests allowed a correct classification at the same performance with an average accuracy of 100% (Table 3). This was achieved, with both the full and reduced feature set emphasizing that machine learning, unlike classical statistics, has no problems in detecting group differences that are not reflected in differences in the central tendency. This is consistent with the effect size estimate of “Impact”, which also indicated effects deviating from zero in the reduced set of variables. In contrast, training the algorithms with permuted data resulted in balanced accuracies of about 50%, as expected, and supports that the perfect classification performance of machine learning algorithms sharply contrasting with the results of the statistical evaluation of the group effects was not achieved by overfitting.

**Table 3:** Performance measures of machine-learning based classifiers applied to date set 4 (Fig 4) with either the full set of variables 1,…,20 or a reduced set [1,…,10,16,…20] from which variables in which the groups differed with respect to their central means [11,…,15] were omitted. Two different machine-learning methods (classification and regression trees (CART) and random forests (RF)) were applied to the artificially generated data set 4, which comprises two groups with sizes of n = 1000 cases and d = 20 variables. The results represent the medians of test performance measures from 100-fold cross-validation runs using random splits of the data set into training data (2/3 of the data set) and test data (1/3 of the data set). In addition, a negative control data set was created by permutating the variables from the training data set, with the expectation that the machine learning algorithms should not perform group assignment better than chance when trained with such data; otherwise, there could be overfitting involved.

	All variables [1,…,20]		Reduced set of variables [1,…,10,16,…,20]		All variables [1,…,20], permuted training data subsets	
	CART	RF	CART	RF	CART	RF
**Sensitivity, recall**	100 (99.1–100)	100 (100–100)	100 (100–100)	100 (100–100)	55.6 (2.8–100)	51.1 (25.2–78.9)
**Specificity**	100 (99.4–100)	100 (100–100)	100 (100–100)	100 (100–100)	48.6 (0–94.9)	51.8 (26.3–72)
**Positive predictive value, precision**	100 (99.4–100)	100 (100–100)	100 (100–100)	100 (100–100)	50 (6.8–93.7)	51.9 (27.5–72.6)
**Negative predictive value**	100 (99.1–100)	100 (100–100)	100 (100–100)	100 (100–100)	50 (11–97.8)	52.1 (29–76.2)
**F1**	100 (99.5–100)	100 (100–100)	100 (100–100)	100 (100–100)	52 (4.7–87.4)	50.9 (26.5–74.7)
**Accuracy**	100 (99.5–100)	100 (100–100)	100 (100–100)	100 (100–100)	50 (17.6–87.1)	52 (28.3–73.7)
**AUC-ROC**	100 (99.5–100)	100 (100–100)	100 (100–100)	100 (100–100)	51.3 (32.5–91.4)	61.4 (50.8–85.6)

#### Robustness of the Impact effect size measure

In settings where “Impact” and Cohen's d show similar effect sizes, as for example for the variable "Var0011" of data set 4 (Fig 4), the robustness of the two measures also compares. In particular, when calculating ten times the effect sizes for data subsets randomly sampled with class-proportional sizes from “Var0011” of data set 4, with fractions decreasing from 100% to 10% of the data (Table 4), the coefficients of variation for both effect size measures increase in a similar manner, but do not exceed about 10% when only 10% of the original data are used. In such an environment, the impact is more likely to reflect its first component, *CTdiff*, and is more likely to be a nonparametric equivalent of Cohen's d. In another setting that is consistent with data set 5, “Impact” indicates a considerable effect size, while Cohen's d indicates no effect. There, “Impact” depends more on its second component, *MorpDiff*, because the central tendencies are the same for both groups in the data. In such a setting, “Impact” is also robust, producing coefficients of variation below 5%, which suggests that both components do not make it a less robust measure than Cohen's d. It is noteworthy that in a setting where Cohen's d is close to zero, its coefficients of variation observed in subsampling settings may inflate (Table 4).

**Table 4:** Parameters of effect size measures obtained for data set 4, Var0011 (means, standard deviations, SD and coefficients of variation, CV) and for data set 5. The experiment was performed in 10 runs with different seeds and using randomly selected 100,…,10% of the original data obtained for samples < 100% by class-proportional random Monte-Carlo [26] resampling from the original data set. The calculations were performed using the “Impact” function of our R library ImpactEffectsize” (https://cran.r-project.org/package=ImpactEffectsize).and the “cohen.d” function of the R library “psych” (https://cran.r-project.org/package=psych [42]), respectively.

	“Impact”			Cohen’s d		
	Mean	SD	CV [%]	Mean	SD	CV [%]
**Data set 4, Var0011**						
**Complete**	2.61	0	0	3.02	0	0
**90%**	2.62	0.014	0.54	3.02	0.01	0.34
**80%**	2.63	0.027	1.04	3.02	0.23	0.77
**70%**	2.64	0.048	1.82	3.04	0.039	1.3
**60%**	2.63	0.52	1.98	3.035	0.047	1.57
**50%**	2.62	0.05	1.89	3.027	0.074	2.44
**40%**	2.54	0.09	3.53	2.96	0.092	3.12
**30%**	2.54	0.105	4.12	2.962	0.114	3.84
**20%**	2.606	0.088	3.4	2.99	0.062	2.06
**10%**	2.623	0.286	10.9	3.03	0.289	9.52
**Data set 5**						
**Complete**	-0.35428	0	0	0.004	0	0
**90%**	-0.35446	0.00109	0.31	0.00464	0.00145	31.29
**80%**	-0.35401	0.00211	0.6	0.00455	0.00152	33.53
**70%**	-0.35426	0.00189	0.53	0.00493	0.00216	43.78
**60%**	-0.354	0.00356	1	0.00321	0.0027	84.37
**50%**	-0.35308	0.00365	1.03	0.00327	0.0033	101.13
**40%**	-0.35168	0.0043	1.22	0.00309	0.00369	119.55
**30%**	-0.35237	0.00501	1.42	0.00382	0.00429	112.2
**20%**	-0.35355	0.00633	1.79	0.00164	0.00722	441.53
**10%**	-0.35228	0.01105	3.14	0.00144	0.01237	856.6

## Discussion

The proposed “Impact” measure captures (treatment) effects or group differences that consist mainly in differences in the form of data distribution and only to a small degree in changes in the central tendency of the data. The proposed measure of effect size is non-parametric, invariant to the scaling of the data, reflects mainly changes in the central tendency in cases where differences in the shape of probability distributions between subgroups are negligible, but captures changes in probability distributions as effects and is numerically stable even if the variances of the data set or its subgroups disappear. By its first and most important property, i.e. the detection of effects on both the distributional form and the central tendency, it meets the needs of effect detection in a biomedical environment where bimodal marker expression is a typical sign of a pathophysiological process [12]. Examples in artificial and biomedical data sets have shown that “Impact” in these cases is more consistent with the scientific observations than alternatives such as Cohen's d as an effect size measure.

“Impact” considers the difference in central tendency as one of its components (*CTdiff*) analogous to Cohen's d with the difference that it takes a non-parametric approach. The calculation of the pooled variability between groups follows a variant also used for Cohen’s d, available as ${SD}_{pooled}=\sqrt{\frac{{SD}_{1}^{2}+{SD}_{2}^{2}}{2}}$. This corresponds to the current implementation in “Impact” of pooled variability of data between groups. Therefore, “Impact” is independent of the size of the two subgroups. This is particularly advantageous if a treated group of patients is small. It is nevertheless noteworthy that typical implementations of Cohen's d would use the pooled standard deviation for two subgroups [37] implemented as ${SD}_{pooled}=\sqrt{\frac{(n_{1}-1)∙{SD}_{1}^{2}+(n_{2}-1)∙{SD}_{2}^{2}}{n_{1}+n_{2}-2}}$. This pooled standard deviation makes the calculation of Cohen's d dependent on the relative sizes of the two subgroups. The standard deviation of the larger subgroup will dominate the pooled standard deviation and is therefore crucial for the pooled central tendency. By mimicking this method of pooled variability with the Gini mean difference (GMD), the scaling of “Impact” indeed gets even closer to Cohen's d.

"Impact" considers the difference in the form of data distribution as a second component (*MorphDiff*). This "morphic difference" considers changes in the form of the distribution. The scaling of these two components was chosen so that the absolute values of the *CTdiff* are unbound, while the *MorphDiff* is between -1 and 1, which means that for large effect sizes the Cohen's d style of effect size measure (*CTdiff*) dominates in the calculation of "Impact". If the effects are small, i.e. the (normalized) central tendency is in a range from -1 to 1, the morphic difference in effect size measure becomes more important. The morphic difference is calculated with (empirical estimates of) probability distributions (*pdf(ξ)*). Estimates of pdf are based on a suitable kernel design, which is as difficult as the pdf-estimation itself [38]. The use of PDE as an estimation aims at detecting even the smallest differences in the shape of the pdf. If a stronger averaging is appropriate, e.g. the use of smoothed data histograms is an alternative [39]. Within these limits, it has been shown that Cohen's d is not able to detect effects (Fig 7 right: Cohen’s d always zero), while “Impact”is proportional to the amount by which the distribution has changed. Impact is a combination of effect on the central tendency and on the form of distribution. So it is perfect to discover "any" effect, for example of a treatment. Occasionally it may be desirable to separate the contributions of both effects, which can be achieved by modifying *CTDiffWeight* (see Eq 1) and is also possible with the R library, which provides both components as separate outputs. Since “Impact” is a non-parametric measure, calculation costs can become a problem. For the "impact", this is dominated by (i) the efficient calculation of the medians (Eq 2) and (ii) the efficient calculation of the Gini mean differences within the data set (Eq 3). For both problems, efficient approaches have been proposed for large datasets [40, 41].

Another potential advantage of “Impact” can be in the processing of large amounts of data in life sciences, which often implies a large number of variables. In such an environment, visual inspection or manual analysis of all variables for their suitability to quantify treatment effects or group differences is not feasible. This increases the need for robust calculations, which are also defined in extreme cases, e.g. when the variances of subsets fall to zero. However, the popular Cohen's d measure for effect size is not defined in this case, and an algorithmic implementation of the Cohen's d would yield unpredictable values. This is not acceptable if a measure of effect size is to be used for the selection of characteristics. Therefore, “Impact” bypasses the limitations of Cohen's d not only as described above by considering broader representations of effects, but also by returning effect sizes where Cohen's d simply does not return a number. On the other hand, by its design that the additional effect introduced by the shape difference in the data distributions is limited to a value of 2 and the effect contributed by the differences in central tendency scales proportionally to Cohen's d with linear increase, the known limits for small, medium or large effects accepted for Cohen's d [36] can be adopted for "Impact".

Effect size measures, if not used for feature selection, are a basis for meta-analyses. If not reported in the original publication, they are usually estimated from the reported measures of central tendency and variance. This can be achieved in a similar way for the proposed measure, i.e. “Impact”can be estimated from the parametric information on the variance. However, this may miss the difference in the shape of the distribution. Ideally, the original data are available that allow the form of the distribution to be estimated, including possible multimodality that is not covered by the standard statistical measures usually reported in scientific papers. A very clear example (Table 2) showed that in some cases typical statistical analyses such as ANOVA with repeated measurements are not able to detect differences in groups where a machine-learned classifier has no difficulty in doing so. Therefore, the proposed effect size measure is directed more towards a data science and machine learning context than statistical data analysis.

## Conclusions

An effect size measure is proposed which, firstly, is robust to any type of data distribution and, secondly, considers the fact that a treatment may have complex effects on the measured characteristics on the central tendency or on the shape of the distribution of the data. However, the established characteristics of Cohen's d remain largely intact when using the newly defined “Impact” effect size measure, which, by its design, allows for a dominance of effects based on differences. Using artificial and real biomedical data sets, it was shown that with “Impact” group differences are better captured than with purely difference-based measures such as Cohen's d. It is also shown that, although classical effect size measures share the failure to observe an effect when the form of data distribution changes, but not in the central tendency with standard statistical analyses, the proposed effect size measures share the ability to observe such an effect with machine learning algorithms. These have no problem in using the shape information for successful classification or group separation tasks. Therefore, the proposed effect size measure is intended more for a data science and machine learning context than for statistical data analysis.
